# The experience of self-advocacy among cancer patients: A qualitative meta-synthesis

**DOI:** 10.1371/journal.pone.0321719

**Published:** 2025-04-16

**Authors:** Lijun Lin, Ying Jin, Chunxiao Feng, Kejia Zhu

**Affiliations:** 1 College of Nursing, Zhejiang Chinese Medical University, Hangzhou, China; 2 Department of Nursing, The second affiliated hospital of Zhejiang Chinese Medical University, Hangzhou, China; Access Alliance Multicultural Health and Community Services: Access Alliance, CANADA

## Abstract

**Background:**

During cancer treatment, patients are faced with major changes in physical function, psychological challenges and decline in quality of life. Self-advocacy is a key tool for patients to cope with the challenges of treatment. By fostering self-advocacy, patients can effectively self-manage, enhancing their overall quality of life and treatment outcomes. Besides, a significant majority of cancer patients encounter barriers when attempting to articulate their healthcare needs and engage in treatment decision-making processes. It’s important to identify obstacles in the process of self-advocacy. The aim of this meta-synthesis was to describe the patients’ experience of self-advocacy, and identify the facilitators and barriers of self-advocacy for cancer patients.

**Methods:**

The review used the Preferred Reporting Items for Systematic reviews and Meta-Analysis (PRISMA) guidelines guided reporting, and appraised the quality of each eligible study using the Critical Appraisal Skills Programme (CASP) checklist. A prospective review protocol was registered in the International Prospective Register of Systematic Reviews(no: CRD42023493926). A qualitative meta-synthesis was performed by searching eight electronic databases, including PubMed, Web of Science, Embase, Ovid MEDLINE, CINAHL, CNKI, Wanfang and SinoMed for studies meeting pre-defined eligibility criteria, from inception to November, 2023. Two reviewers independently undertook screening and review of articles, using the CASP checklist for evaluating qualitative research. The data were synthesised using Thomas and Harden’s method of thematic and content analysis.

**Results:**

A total of 7 papers were included, and 24 research findings were distilled and integrated into three themes: benefits; challenges; external environmental support; and seven sub-themes: Gain confidence; improve self-management ability; Interaction and share; lack of awareness; obstacles; health system support and social support.

**Conclusions:**

Cancer patients have different levels of self-advocacy ability, which is the result of the interaction between personal consciousness and family and social environment. Factors influencing self-initiative include patient gender, personality characteristics, support from friends and family, and support from the medical system. Therefore, medical staff should pay more attention to cancer patients with weak awareness of self-advocacy and poor enthusiasm and can use patient friend exchange meetings and entertainment interventions to improve patients ‘level of self-advocacy. Future interventions should comprehensively consider the characteristics of cancer patients themselves and their external environment, and engage in multidisciplinary team cooperation.

## Introduction

In January 2021, cancer data released by the International Agency for Research on cancer (International Agency for Research on Cancer, IARC) of the World Health Organization showed that there were 19.3 million new cases of cancer and almost 10 million deaths in the past year, and global cancer burden is expected to be 28.4 million cases in 2024 [1]. The continuous development of cancer diagnosis treatment, and care systems, the constant spread of patient-centered concepts, and the transformation of acute care to chronic care make cancer care more complex and the needs of cancer patients more diverse [2–4]. Research showed that self-advocacy is a means of improving patient-centered care capabilities and a crucial tool in responding to cancer treatment [5,6]. The concept of self-advocacy first originated from the study of the moral and legal rights of adolescents, people living with AIDS and people with disabilities. In 1970, the Candlelighters Children’s Cancer Foundation was established and advocated for child cancer survivors and their families [7]. In the HIV population, Brashers *et al.* [8] defined self-advocacy as patients taking a participatory position in healthcare interactions and becoming‘activists’ in managing their illness. In the disability population, Vessey *et al.* [9] defined the concept as ‘the ability to seek, evaluate and use information to promote one’s own health’. In 1996, Clark and Stovall [10] first put forward the concept of self-advocacy for cancer patients, and summarized it as a series of skills necessary to overcome cancer, such as information gathering, communication, problem-solving and negotiation skills. As self-advocacy becomes widely used in other patients, more research has proven that it is a trainable skill that can improve patients ‘quality of life and health outcomes [11,12]. In 2013, through conceptual analysis, Hagan and Donovan [13] summarized the right to self-advocacy into three aspects, including seeking or providing support, sharing their cancer experiences, and raising people’s awareness of cancer. This provided a clear concept of the development of self-advocacy among cancer patients. In 2020, *Abbasinia et al.* [14] extracted a new definition of self-advocacy from 46 articles and 2 books, arguing that self-advocacy is a dynamic concept, not just support, care and compassion. Patient self-advocacy includes the protection of health care, knowledge, attention, mediation and the promotion of social equity in health care. In 2021, *Thomas et al.* [5] defined self-advocacy as the ability of an individual with cancer to overcome challenges in getting their preferences, needs, and values met. This provided a direction for the further development of self-advocacy of female cancer patients.

Cancer patients need to improve their self-care capabilities to cope with the challenges posed by cancer due to high mortality, heavy symptom burden, aggressive pathological changes, uncertainty of the disease, and fear caused by high recurrence rates. Research showed that the level of self-advocacy is closely related to whether cancer survivors can reflect their personal needs, priorities and values, and receive patient-centered care [15]. Moreover, self-advocacy helps patients actively participate in treatment decisions, understand the disease, compare the risks and benefits of multiple cancer therapies, and finally follow the heart to choose the right plan [13]. Moreover, the research showed that self-advocacy can help cancer survivors develop a strong sense of self-awareness, self-control and adaptability to cancer. At the same time, promote its initiative to collect disease information, obtain external support and establish a tight link with the medical team to meet its own needs, and improve its life quality [5,6].

Although positive results of self-advocacy have been reported in the current study, there are many obstacles to patient self-advocacy. Research showed that challenges such as lack of confidence, limited health knowledge, or insufficient social support can hinder patients from receiving treatment [16]. There has been no prior published review of qualitative studies in this area; therefore, we implemented a systematic review to explore and synthesize the existing qualitative research findings to comprehensively determine the experiences of self-advocacy among adults patients with cancer, so as to better meet the needs of patients and provide a basis for high-quality realization of self-advocacy.

## Methods

A qualitative meta-synthesis was performed to explore cancer patients’ perspectives of self-advocacy. The revised Preferred Reporting Items for Systematic Reviews and Meta-Analyses (PRISMA) 2020 checklist [17] was followed to report the findings. The review protocol was registered in the International Prospective Register of Systematic Reviews (no: CRD42023493926). The completed PRISMA checklist is available in S1 Appendix. The reporting of this meta-synthesis adhered to the ENTREQ guideline [18](see S2 Appendix).

### Design

Meta-synthesis is a rigorous research method used to analyze and synthesize the results of existing qualitative research and construct high-order meanings by identifying overall themes [19].

This study used Thomas and Harden’s theme analysis method [20]. Through the analysis of the content characteristics of the literature, the subject concepts are extracted, and the significance and essence related to the research are analyzed and summarized. The process of data extraction and integration includes coding, creating descriptive theme and constructing analytical theme.

### Database and search strategy

We searched PubMed, EMBASE, Web of Science, MEDLINE, CINAHL, SinoMed (China), Chinese Citation Database (CNKI), and Wanfang Data (China) in November 2023, and updated the search on 10 November 2023. After preliminary testing, three key concepts were finally identified, including self-advocacy, tumor, and qualitative research. Keywords/search terms were extracted from the three key concepts. Then we used Medical Subject Titles (MeSH) and online synonyms to search for synonyms that derive the keywords. We used a combination of subject terms and free words as search strategies. Additionally, we included relevant references from the identified studies through a retrospective approach. S3 Appendix shows search terms in full, including free text and MeSH terms.

### Selection: Inclusion and exclusion criteria

Literature screening based on the established acceptance standards(see Table 1).

**Table 1:** Inclusion and exclusion criteria.

	Inclusion	Exclusion
Study type	Qualitative research, or mixed methods research (extracting only the qualitative component), includes the use of phenomenological research, grounded theory and other methods.	Existing qualitative meta- syntheses or reviews; Duplicate publications, Conference abstracts, PhD theses, books, commentaries, dissertations or other types of grey literature
Population	Adult cancer patients(Aged 18 and above, having any type of cancer)	Studies including qualitative data collected only from healthcare providers and partners or other individuals with close experience of interaction with cancer patients
Concept	At least part of the results section concerned participants’current or retrospective experi- ence of self-advocacy	Papers presenting data of self-advocacy incompletely or not at all
Context	Studies in any geographical or cultural settings	
Language	Chinese and English	Not in Chinese or English

### Data screening and extracting

The retrieved literature was imported into EndNote X9, and duplicates were removed by software check. The titles and abstracts of all included literature were screened by author 1 (LJL) based on inclusion criteria. Author 4(KJZ) independently screened and randomly selected 10% of titles and abstracts to check consistency. author1(LJL) and author3(CXF) then read the full text of all potentially eligible papers and, after discussion, finally determined the included literature.

We developed a data extraction proforma, which included: author, country, year, research purpose, aims, sample size, analysis method, and main results. Data extraction was completed by author 1, and author 2 checked the accuracy. Any discrepancies were resolved through discussion until consensus was reached.

### Data analysis and synthesis

Data analysis was divided into three stages: (1) Two researchers analyzed the included literature. After extracting the relevant content of “cancer patients ‘experiences of self-advocacy” in the literature results section, the two researchers analyzed the extracted relevant content verbatim; (2) Another study compared and integrated the descriptive theme proposed by the two researchers previously to form the final descriptive theme; (3) After one researcher repeatedly thought and discussed the descriptive theme, more abstract topics began to appear. Based on these topics, the research team conducted further discussions and reviews to determine the final analytical theme. All analyses were performed using Word and Excel files.

### Quality appraisal

The Critical Appraisal Skills Programme Qualitative Studies Checklist [21] was applied by two authors to access the quality the included studies independently. The evaluation content involves a total of 10 aspects, and each item is evaluated as “yes”, “no”, “unclear” and “not applicable”. If any differences arose, they were discussed and sent to a third reviewer (if unresolved after discussion) to obtain a consensus. Although one included study scored only 7 points, considering the small number of research literature, it was finally decided to include it. Detailed quality appraisal results are described in S7 Appendices.

## Results

### Study characteristics

**Fig 1:**
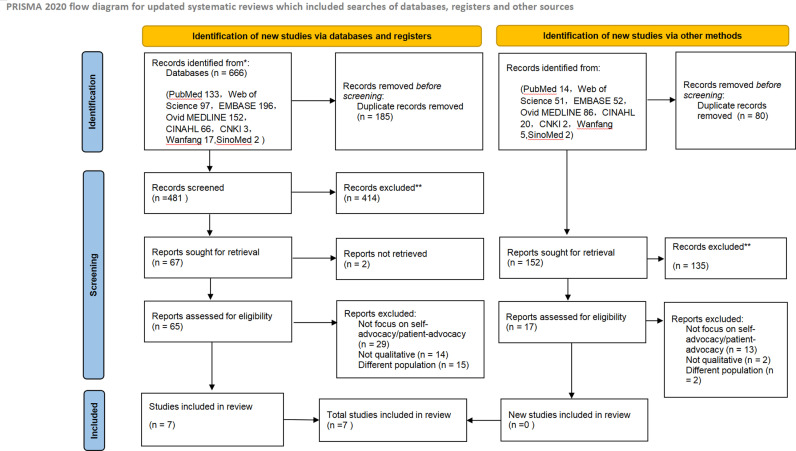
PRISMA flow diagram for included studies.

The search identified 666 papers of which 185 were duplicates, and 414 citations were excluded after title and abstract screening. A total of 67 studies were reviewed in full, and 60 articles were excluded at this point for the specific reasons. Our search updated on February 5, 2025, found no studies that met the criteria included. In total, 7 studies were included in this review (see Fig 1). All research was published between 2013 and 2023, with six studies done in America [22–27] and the other done in China [16]. Table 2 describes the features of the included studies.

**Table 2:** Characteristics of included studies.

Study	Aim	Demographic		Methods			Results
		N	Sample	Methodology	Data collection	Data analysis	
Sarah Bell et al(2023)	Describe the self-advocacy experiences	10	advanced breast or gynecologic cancer	descriptive qualitative study	semi-structured interview	Content analysis	speaking up and speaking out; interacting with the healthcare team; relying on support from others
Hagan et al(2013)	Explore ovarian cancer survivors’ experiences of self-advocacy	13	ovarian cancer survivors	phenomenon study	in-depth interviews	Content analysis	knowing who I am and keeping my psyche intact; knowing what I need and fighting for it.
Thomas et al(2022)	To describe the key components of self-advocacy among men with cancer.	28	adult men with a history of invasive cancer	descriptive qualitative study	semi-structured interview	Content analysis	managing through information and planning; finding the best team and falling in line; strategic social connections
Thomas et al(2023)	The participant perspectives of a novel, self-advocacy serious game intervention	40	women with advanced cancer	qualitative study	one-on-one interviews	Content analysis	1.overall acceptability; 2. seeing myself in most scenarios and wanting more content; 3. giving me the go ahead to expect more; 4. offering ideas for how to stand up for myself; 5. reinforcing what I am already doing; 6. reminding me of what I have.
Sydney et al(2017)	To engage survivor–advocates by describing their experiences living with lung cancer	19	patients with lung cancer	qualitative study	one-on-one interviews	Narrative analysis	Stage I. Live: The stage “Live” refers to survivors’ personal lived experience with lung cancer themselves; Stage II. learn: Knowledge gives me a future addresses the importance of accessing knowledge and ways to approach this; Learning to be empowered in my care; Finding my way through the system; Stage III. pass it on: Pulling lung cancer out of the shadows; The urgency of sharing stories; Showing the way involved becoming a guide to other patients; The secret handshake of community was also vital
Hagan et al(2016)	To explore the language of self-advocacy	13	woman cancer Survivors	qualitative study	focus group	Discourse analysis	maintaining a positive attitude needing and being scared of information connection with health care team
Zhirong Jiang et al(2023)	To gain an in-depth understanding of the self-advocacy experience of breast cancer patients	20	woman with breast cancer	phenomenon study	semi-structured interview	Colaizzi’s Method	weak awareness of self-advocacy multiple factors on self-advocacy motivation challenges in the process of self-advocacy positive experience of self-advocacy

### Methodological quality

The evaluation of each study’s quality is presented in S7 Appendix. The CASP checklist scores of the papers ranged from 7 to 10. Most studies clearly stated the research aims and results and used qualitative study designs appropriately. However, the CASP results showed that the researcher and patient relationship was not elaborated in most included studies (85.7%), reducing the study quality. The description of the research results in the included studies conformed to the specifications, and the overall quality was above medium and less affected.

### Meta-synthesis

**Fig 2:**
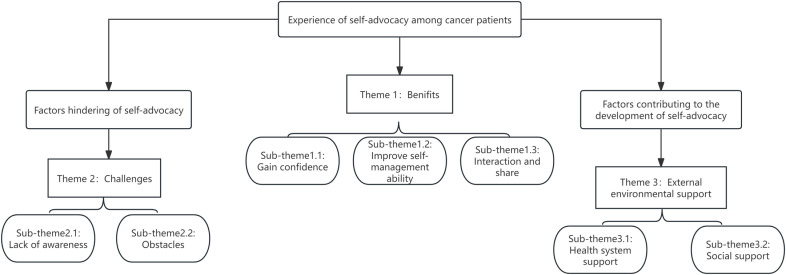
Map of meta-themes and sub-themes.

This study synthesised the results of a qualitative study of 144 cancer patients, generating three themes (benefits, challenges and external environmental support) and seven sub-themes (gain confidence, improve self-management ability, Interaction and share, lack of awareness, obstacles, health system support and social support). Fig 2 represents the thematic framework.

#### Theme 1. Benefits.

In most studies, the positive experience of self-advocacy was the most frequently mentioned feeling by cancer patients. It improved patients’ confidence and self-management ability.

***Sub-theme 1.1: Gain confidence:*** Several studies(researches from America and China) showed that cancer patients gained confidence to facing disease through self-advocacy. In the past, patients did not know self-advocacy was acceptable or even desirable by their healthcare providers.*‘I never knew that I could interrupt a doctor to ask a question, to add a new direction to my care.’* [25]*.* Most cancer patients formed a positive attitude to deal with everything related to cancer.*‘*Strong will would be the word. Because no matter what … we’re just fighting to survive.*’* [23]*‘it allows her to fight, keep a sense of self worth, and believe that she can overcome the cancer and “never go down.”’* [27]*.‘I also like to say to patients, like the ones we played well in it, I did a lot of ideological work with them. All of us added Wechat, so we said that we had this disease and faced it bravely together’* [16]*.* Patients obtained other treatment plans through many ways, and got good outcomes, and were full of hope for their future life.*‘I had been hearing online from people throughout the year about a new mutation called ROS1... I gave them permission to test... I had a very, very strong indication of a ROS1 mutation. Three days later I’ve flown out [of state]. I took my first pill... And my very first scan there was no evidence of disease and have been for 28 months.’* [26] With the deepening of the process of self-advocacy, patients also had further requirements.*‘They had issues that I am not experiencing. But at the same time, I may down the road. So, it was good to know what might be coming as far as other people’s experiences’* [25]*.*

***Sub-theme 1.2: Improve self-management ability:*** Cancer patients received lots of advice from healthcare providers, advocacy organizations, friends, and the Internet. They discovered how to filter and manage the information to meet their needs and to achieve their goals.*‘It’s comforting. It helps you decide things about whether to go out, whether to run, whether to go shopping, call your doctor, all of the above. So, for me, it was like an outline of’* [25]*.* Cancer patients not only learned disease knowledge from others, but also take the initiative to find ways to acquire relevant knowledge.*‘They’re going to put me on this new inhibitor program and I’m reading more about it; I got all the details on it.’* [23]*.* Moreover, self-advocacy helps patients face reality rationally, rather than facing cancer with an evasive attitude. Male cancer patients want to get the truth from their doctors.*‘Stop telling patients lower percentages about side effects, recurrences, and watchful waiting. When all else fails, tell the truth!’* [28]. However, not all patients liked to take the initiative to obtain information, and some patients trusted the information from health providers more [27]. After cancer patients form a sense of self-advocacy, they will express their demands and make decisions more bravely.*‘ if I find a medication isn’t helping me, then I do speak up, OK? Now in that way, yeah, I do do that.’* [22]*‘I approach my doctor and tell him what I’m thinking and get his feedback, if he disagrees, and so I advocate for what I feel is the best treatment available.’* [26] In addition, patients also knew more clearly what they should do.*‘Any health issues, I get up there right away whether it’s a pain in my back, or you know, something with my eyes or whatever, you just [say].’* [23]*‘It kind of reinforced things, what you should or should not do.’* [25] For male patients, maintaining a focus on treatment can help them control and dismiss their negative thoughts.

***Sub-theme 1.3: Interaction and share:*** Cancer patients shared their self-advocacy experiences with others and further share their experiences.*‘I did a lot of ideological work for several patients who were playing well’* [16]*‘Wherever I can—I feel somebody that has survived— somebody that has lived longer than the average, I feel obligated in my role to stand up and speak from as high a perch as I can possibly speak.’* [26] Cancer patients not only share their own stories but also actively guide other patients to advocate themselves.*‘It’s really about trying to get those people out of the dark or out of the loneliness of the disease so that they can be advocates.’* [26] In addition, patients also actively participated in local and national organizations, disseminated important information about related diseases through the media, shared personal stories, and changed people’s views.‘I’m hopeful that there will be a true outpouring, nationwide, of compassion for people with lung cancer, like an awakening so to speak, enlightenment, of, “Hey, these people do matter”.’ [26] Moreover, most patients thought that management skills, educational skills, public speaking ability and excellent writing skills are beneficial to spread self-advocacy [28].

#### Theme 2. Challenges.

In some studies, patients also faced some obstacles and challenges in the process of self-advocacy.

***Sub-theme 2.1: Lack of awareness:*** In the course of cancer treatment, some patients lacked the consciousness to report their symptoms actively. They thought symptoms are normal and didn’t need to talk to the doctor.*‘I was bored and uncomfortable after chemotherapy. That is to say, they (patients) responded after a drip. I didn’t tell the doctor that I had to overcome it slowly by myself. This is all a response to chemotherapy. Is there any way to solve it ?(sigh)’* [16]*.* Affected by the professionalism of doctors, some interviewees thought that when you come to a hospital, you only need to follow the doctor’s advice and lacked a sense of autonomy.*‘Now that I have a disease, the doctor says it is better to treat it. I have never had this disease, and I do not know whether the treatment plan is good or not. He said that this plan is good, so let’s follow it.’* [16]*.* Cancer patients didn’t have the awareness to participate in medical decision-making actively, only focused on their discomfort symptoms, and lacked attention to other needs.

***Sub-theme 2.2: Obstacles:*** The obstacles of patients’ self-advocacy came from their own factors, family factors, medical staff factors and economic conditions. Patients had an evasive mentality and a sense of shame and they were afraid to report symptoms for fear of increasing the cost of treatment. *‘ I think releasing that responsibility and putting it on a physician, somebody that’s knowledgeable, is a relief.’* [22]*‘Now that I have had chemotherapy, my hair has fallen out and my breast has been cut off. I feel very inferior. I don’t want to deal with others, nor do I want to tell anyone about me.’* [16]. Under the influence of Chinese traditional thinking, family members thought that it is not necessary for patients to understand disease-related information and didn’t support patients to seek health information.*‘*It was all my children who went to communicate with the doctor, but they didn’t tell me. My two children said you didn’t need to listen to these things and told me not to inquire about them.*’* [16] Some medical staff lacked the concept of “patient-centered” medical care, and held a negative attitude towards patients’ needs.*‘*During the ward rounds yesterday morning, I wanted to ask a few questions, but the doctor said directly: ‘Don’t talk about anything else. You just need to answer my questions. You can only answer yes or no.’ I am speechless.*’* [16] The possible reason is that medical staff have heavy clinical work, have no time to take into account the inner needs of patients, and lack accessible and convenient self-advocacy educational resource.

#### Theme 3. External environmental support.

Multiple external support is more conducive to patients’ self-advocacy.

***Sub-theme 3.1: Health system support:*** Health system support played a key role in the process of patients’ self-advocacy. Health system support leads patients to face cancer honestly.*‘I need her guidance, but I just also need her to be completely honest with me about what’s happening, and she’s doing that.’* [22]. *‘Three participants cited honesty from their care team as being a key component to good care.’* [22] Not only nursing support but also the support of the entire healthcare team is critical to patients.*‘care from*

them, from the whole team, from my doctor to the nurses to everybody in the office.’

However, different patients had different degrees of trust in their health care providers.*‘Even though Colleen fully trusts her provider and Dorothy has learned to be more skeptical’* [27]. In addition, when choosing a health care provider, male patients gave priority to knowledge and implementation, rather than being friendly or approachable [24]. Based on cultural background and clinical reality, it was further found that in China, patients believe that their relationship with doctors is like students and teachers. They must be obedient patients, dare not raise their doubts, and rely more on the medical team [16]. Patients in the United States prefer to put forward their own needs and choose a medical team they trust [22,24].

***Sub-theme 3.2: Social support:*** In addition to the support of health care providers, patients also received support from family, friends, employers, and community.*‘I did need her voice and her ears and her eyes and her strength. Because we have no other family in the area, so it’s just my husband and I.’* [22]*‘Believe me, my employer has been more than generous and willing to assist me, particularly with doctors’ appointments that have come up unexpected’* [22]*”But people came surprisingly up to me when I was first diagnosed to let me know, ‘We’re here, and we can go with you to get a wig. Don’t worry. We’ve all been through this.’ People that I never even knew had breast cancer that told me other people that I never knew either that had breast cancer. So I’m not lacking in a social network.”* [22] Male cancer patients said they were worried that their partners would have a negative reaction if they knew about it and affect their ability to support their families. Hence, male patients carefully considered who they will consider when discussing their cancer problems and purposefully accepted the support of others. Although many men say they prefer to deal with problems themselves, they feel an unprecedented sense of gratitude and connection when friends or extended family reach out to support them [24].

## Discussing

This study systematically reviewed and integrated 7 qualitative studies to summarise the experiences of self-advocacy among cancer patients. Data synthesis led to the construction of three analytical themes: Benefits; Challenges and External environmental support, which contribute theoretical understanding to the experience of self-advocacy.

### Pay attention to the positive power of self-advocacy and expand the scope of application

According to the results of this study, self-advocacy can build patients ‘confidence, help them understand cancer with a more positive attitude, proactively collect relevant data, dare to express their demands, and thus choose appropriate treatment plans according to their own heart [16,22,25,27]. The findings of this review highlight that self-advocacy facilitates symptom management, promotes mental health, improves quality of life, and facilitates patient participation in medical decision-making for cancer patients. Our findings are also consistent with some quantitative studies [11,29,30]. Simply knowing how to manage and live with their symptoms was not enough for patients; they had to internalize those abilities and, in so doing, infuse their personal values and beliefs into their symptom management processes. We need to pay attention to the positive experience of patients’ self-advocacy and stimulate the inner strength of the individual. In the process of self-advocacy, patients not only improve their self-management abilities and gain self-identification but also guide other patients to self-advocacy by sharing their experiences, and passing on positive power to others while also gaining accomplishment, forming a virtuous cycle. As a result, patients ‘sense of self-efficacy has been improved, and self-advocacy has been further promoted [16]. Research showed that organizing regular patient exchange meetings can help patients gain skills, and emotional and information support and is one of the important ways to spread positive power [31,32]. It’s recommended that hospitals set up special exchange meetings for self-advocacy patients to enhance patient enthusiasm and expand the scope of the application of self-advocacy. At present, the scope of self-advocacy is limited. Most of the literature included in this review comes from the United States, and it is recommended to be applied in other countries and regions in the future to obtain more experience and evidence.

### Reduce obstacles and optimize the way of participation

The findings of this study emphasize that there are also obstacles to the self-advocacy process, including patient, family, and medical system factors. However, affected by gender, personality, education level, and cultural differences, patients’ feelings of self-advocacy are different. Some patients fully trusted her provider, others had learned to be more skeptical [27]. This study found that the awareness of self-advocacy of patients from rural areas, low education level and old age was weaker [16]. In fact, these findings are congruous with the results from quantitative studies. *Kolmes et al.* [33] showed a study indicated that the level of self-advocacy was higher in women than in male patients. The probable reason for this is that female patients are better at expressing themselves, whereas males get in the way and lack expression of their claims. There are other reasons that some men concerned that their partners may have negative reactions and impacted their ability to support their families [24]. In addition, a survey showed that patients who are outgoing, confident and have a strong sense of self-awareness and responsibility are more likely to achieve self-advocacy [34]. Studies showed that black female cancer patients tend to remain silent about their concerns [35,36]. In addition, in the social and cultural context of China’s “family-oriented”, family members are worried that patients will learn more about the disease, which will increase their ideological burden and require medical staff not to tell them, resulting in patients lacking access to knowledge [16].

In addition, the professional level of medical staff, attitude towards patients, and relationship with patients are also important factors in self-advocacy [16,27,37]. Research showed that some medical staff has a weak sense of “patient- centered” [16]. If the medical staff doesn’t proactively inform the patient, the patient doesn’t even know that he has the right to self-advocacy. Influenced by Chinese traditional thought, patients believed that questioning doctors during treatment meant cooperating with doctors, and relied more on the medical team for fear of damaging the doctor-patient relationship [16]. The impatient and perfunctory attitude of the medical staff aggravated the patient’s nervousness and fear, and they dared not express the patient’s demands. However, the implementation of self-advocacy also conflicts with the busy clinical work and the lack of easily accessible self-advocacy resources. Studies showed that interventions through entertainment aids and serious games can encourage patients to actively express their wishes and improve their level of self-advocacy [29,38,39]. It’s suggested that in the future, more large-sample, multi-controlled long-term clinical trials can be carried out through recreational methods to provide more reliable evidence for the implementation of self-advocacy. In addition, oncology nurses play an important role as patient advocates in promoting patient self-advocacy [15]. Oncology nurses should encourage patients and their families to actively participate in healthcare decision-making to meet their preferences and needs. It’s recommended to increase the clinical medical staff’s attention to self-advocacy, evaluate patients ‘level of self-advocacy, and provide more support to patients with low levels of self-advocacy.

### Strengthening social awareness and improving support systems

On the one hand, the encouragement, companionship and support of family and friends are the spiritual support for patients to live bravely. It is suggested that medical staff should encourage patients to communicate with their family and friends to express their ideas and needs, so as to get more help. Male patients, in particular, were worried that their partners, jobs, and social status would be affected, so they dared not truly express their thoughts and needs [24]. At the same time, medical staff should pay attention to the positive impact of self-advocacy on cancer patients and create an equal and open doctor-patient communication atmosphere. This requires medical staff to continuously improve their professional skills, actively communicate, and listen patiently. On the other hand, self-advocacy organizations and support systems are an important way for cancer survivors to access health information and resources, as well as an important means of promoting self-advocacy among survivors. Research has developed a self-advocacy training program, the Cancer Survival Toolbox, to teach cancer survivors how to self-advocacy to meet their needs and preferences [40]. The American Cancer Society, the American Institute for Cancer Research, the American Society of Clinical Oncology, and cancer care organizations have established support systems that provide free counselling, education, or financial assistance to cancer survivors [41]. Schear et al. [42] also pointed out that there is a need to establish multi-party organizations, including patients, healthcare professionals, public interest organizations and the media, and to organize cancer forums or experience-sharing sessions on advocacy strategies and planning, in order to improve attitudes, knowledge, policies and services, and to meet the needs of cancer patients at the individual, social and national levels, so as to improve the level of their self-advocacy. In addition, special breast cancer advocacy groups have been set up, such as Y-ME, Susan G. Komen Breast Cancer Foundation and the National Breast Cancer Alliance, which have raised the provision of breast cancer information and support to the political level [43]. It also provided a reference for other countries to establish support system to other cancer patients. With the development of information technology, patients with high e-health literacy will actively search for online information to obtain disease-related knowledge and use it for disease self-management. It’s recommended to use the advantages of the Internet to better help patients manage themselves, such as decision-making tools and electronic self-symptom reporting procedures [44].

This study summarized patients’ experiences of self-advocacy, which is currently limited in scope and is hoped to be promoted in various healthcare institutions in the future to facilitate cancer patients’ symptom management, psychological health, quality of life, and patients’ participation in medical decision-making. At the same time, we should design a variety of self-advocacy methods to increase the interest of patients’ participation, and conduct a large-sample randomized controlled study by combining the existing self-advocacy assessment tools to dynamically record the changes in the process of patients’ self-advocacy and to observe the effects of its application.

### Strengths and limitation

This systematic review utilised rigorous and standardised methods for appraisal, and inclusion is supported by a systematic approach. Qualitative studies of cancer patients and particularly self-advocacy experiences is a sparsely investigated area. however, through careful appraisal, seven articles were found that could be included in this meta-synthesis. The studies represented patients from various countries and cultural backgrounds providing a richer description of this population’s experience.

The findings of this review must be interpreted within its limitations. Firstly, due to the language restriction, this review only examines the contributions in Chinese and English, which can make the results of the study exist in a certain bias. Secondly, personal subjective bias can’t be completely avoided in the study, which may have an impact on the interpretation of integration results. The third limitation is that original data was not accessed, and therefore, any original bias in the primary studies could have led to bias in the synthesis. Finally, the majority of the included studies were from the US. Cultural variation may impact the experiences of the self-advocacy. Therefore, the findings can be generalized only to places with similar contexts. More studies need to be conducted outside the United States.

### Implications

Cancer patients planning to initiate self-advocacy can be supported by the results in this meta-synthesis. Participants in the included studies reported mainly positive effects includes gain effective knowledge, enhance self-management ability, and improve the quality of life. However, we also report on the challenges cancer patients face in the process of self-advocacy, including own factors, family factors and medical staff factors. We believe that self-advocacy may be helpful for cancer patients in relation to undergoing self-advocacy to ease the adjustment to physical and mental changes. Further research to improve the level of cancer self-advocacy and to improve the intervention measures to support self-advocacy are needed.

## Conclusion

Understanding the self-advocacy experiences of cancer patients may help raise awareness of the problem, our study offers evidence for understanding self-advocacy from the patients’ perspective and adds supporting information for improving the level of patients’ self-advocacy. The results reveal that in the process of self-advocacy, cancer patients learn to deal with disease-related problems with a positive attitude, and take the initiative to express their own ideas and participate in decision-making with health care providers. At the same time, some patients encounter obstacles in the process, including differences from the patient’s own, family, medical system, and cultural background. It’s recommended that medical staff encourage patients to express their needs and provide psychological and information support to create a good doctor-patient communication atmosphere. Future research can develop multi-level and multi-form culturally appropriate intervention measures for cancer patients, family members, medical service providers, communities, and the public themselves based on the characteristics of different groups of people to maximize the level of self-advocacy of cancer patients. In the future, large-sample and long- term research are also needed to verify the long-term impact of self-advocacy.

## Supporting information

S1 Appendix PRISMA checklist. (DOCX)

S2 Appendix ENTREQ checklist. (DOCX)

S3 Appendix Search strategy. (DOCX)

S4 Appendix Table1 Inclusion and exclusion criteria. (DOCX)

S5 Appendix List of studies excluded at full text screening. (DOCX)

S6 Appendix Table 2 Characteristics of included studies. (DOCX)

S7 Appendix Results of CASP quality appraisal. (DOCX)

S8 Appendix Themes, sub-themes, original descriptive theme, and illustrative quotations. (DOCX)

S1 Fig PRISMA 2020 flow diagram for updated systematic reviews which included searches of databases, registers, and other sources. (TIF)

S2 Fig Relationships between themes. (TIF)
