# Supplementary material for: The experience of self-advocacy among cancer patients: A qualitative meta-synthesis
Source: PLoS One. 2025 Apr 16;20(4):e0321719. doi: 10.1371/journal.pone.0321719 (PMC12002448; doi:10.1371/journal.pone.0321719)
Supplement: S3 Appendix — (DOCX) [file pone.0321719.s003.docx]

**S3 Appendix: Search Strategy**

| Database | Search strategy | Original Result | Updata rusult |
| --- | --- | --- | --- |
| PubMed | (((self-advocacy[Title/Abstract]) OR (patient-advocacy[MeSH Terms])) AND (((((neoplasms[Title/Abstract]) OR (cancer[Title/Abstract])) OR (tumor[Title/Abstract])) OR (oncology[Title/Abstract])) OR (carcinoma[Title/Abstract]))) AND (((((((experience[Title/Abstract]) OR (qualitative study[Title/Abstract])) OR (qualitative research[Title/Abstract])) OR (phenomenological study[Title/Abstract])) OR (focus groups[Title/Abstract])) OR (grounded theory[Title/Abstract])) OR (mix method[Title/Abstract])) | 133 | 14 |
| Web of science | TS=(self-advocacy OR patient-advocacy) AND TS=(neoplasms OR tumor OR cancer OR oncology OR carcinoma) AND TS=(experience OR qualitative research OR qualitative study OR phenomenological study OR focus groups OR grounded theory OR mix method) | 97 | 51 |
| EMBASE | 1.(self-advocacy or patient-advocacy).ab,ti.  2.(neoplasms OR tumor OR cancer OR oncology OR carcinoma).ab,ti.  3.(experience OR qualitative research OR qualitative study OR phenomenological study OR focus groups OR grounded theory OR mix method).ab,ti. | 196 | 52 |
| MEDLINE | #1“self-advocacy”[Topic] OR ”patient-advocacy”[Topic]  #2”neoplasms”[Topic] OR ”tumor”[Topic] OR ”cancer”[Topic] OR ”oncology”[Topic] OR ”carcinoma”[Topic]  #3”experience”[Topic] OR ”qualitative research”[Topic] OR ”qualitative study”[Topic] OR ”phenomenological study”[Topic] OR ”focus groups”[Topic] OR ”grounded theory”[Topic] OR ”mix method”[Topic]  #1 AND #2 AND #3 | 152 | 86 |
| CINAHL | S1(MM”patient-advocacy”) OR (MM”self-advocacy”)  S2(MM”neoplasms”) OR (MM”tumor”) OR (MM”cancer”) OR (MM”oncology”) OR (MM”carcinoma”)  S3(MM”experience”) OR (MM”qualitative research”) OR (MM”qualitative study”) OR (MM”phenomenological study”) OR (MM”focus groups”) OR (MM”grounded theory”) OR (MM”mix method”)  S1 AND S2 AND S3 | 66 | 20 |
| CNKI | Theme(self-advocacy) AND Theme((neoplasms OR tumor OR cancer OR oncology OR carcinoma)) AND Theme((experience OR qualitative research OR qualitative study OR phenomenological study OR focus groups OR grounded theory OR mix method)) | 3 | 2 |
| WANG FANG DATE | Theme(self-advocacy) AND Theme((neoplasms OR tumor OR cancer OR oncology OR carcinoma)) AND Theme((experience OR qualitative research OR qualitative study OR phenomenological study OR focus groups OR grounded theory OR mix method)) | 17 | 5 |
| SinoMed | (self-advocacy) AND Mesh(tumor) AND ((experience OR qualitative research OR qualitative study OR phenomenological study OR focus groups OR grounded theory OR mix method)) | 2 | 2 |
