# Supplementary material for: The experience of self-advocacy among cancer patients: A qualitative meta-synthesis
Source: PLoS One. 2025 Apr 16;20(4):e0321719. doi: 10.1371/journal.pone.0321719 (PMC12002448; doi:10.1371/journal.pone.0321719)
Supplement: S4 Appendix — (DOCX) [file pone.0321719.s004.docx]

**S4 Appendix:Table 1. Inclusion and exclusion criteria.**

|  | Inclusion | Exclusion |
| --- | --- | --- |
| Study type | Qualitative research, or mixed methods research (extracting only the qualitative component), includes the use of phenomenological research, grounded theory and other methods. | Existing qualitative meta- syntheses or reviews;Duplicate publications,Conference abstracts, PhD theses, books, commentaries, dissertations or other types of grey literature |
| Population | Adult cancer patients(Aged 18 and above, having any type of cancer) | Studies including qualitative data collected only from healthcare  providers and partners or other individuals with close experience of interaction with cancer patients |
| Concept | At least part of the results section concerned participants’current or retrospective experi- ence of self-advocacy | Papers presenting data of self-advocacy incompletely or not at all |
| Context | Studies in any geographical or cultural settings |  |
| Language | Chinese and English | Not in Chinese or English |
