# Supplementary material for: The experience of self-advocacy among cancer patients: A qualitative meta-synthesis
Source: PLoS One. 2025 Apr 16;20(4):e0321719. doi: 10.1371/journal.pone.0321719 (PMC12002448; doi:10.1371/journal.pone.0321719)
Supplement: S5 Appendix — (DOCX) [file pone.0321719.s005.docx]

**S5 Appendix: List of studies excluded at full text screening**

| N | **Item** | **Screen on Full Text** |
| --- | --- | --- |
| 1 | Bellomo(2019)  (ID: /) | -EXCLUDE on "Conference abstracts, PhD theses, books, commentaries, dissertations or other types of grey literature"  Conference abstract has no PMID |
| 2 | Calderone(2021)  (ID:/ ) | -EXCLUDE on "did not include population with cancer"  The literature is from embase and is not included in PubMed, so there is no PMID |
| 3 | Carrasco(2021)  (ID: 34123474) | -EXCLUDE on "did not focus on self-advocacy experience" |
| 4 | Charalambous(2008)  (ID: 18845478) | -EXCLUDE on "did not focus on self-advocacy experience" |
| 5 | Connors(2021)  (ID: 33888195) | -EXCLUDE on "did not focus on self-advocacy experience" |
| 6 | Cowan(2019)  (ID: 31335249) | -EXCLUDE on "did not focus on self-advocacy experience" |
| 7 | Crooks(2001)  (ID: 11813800) | -EXCLUDE on "did not focus on self-advocacy experience" |
| 8 | Dave(2023)  (ID: 37346994 ) | -EXCLUDE on "did not include population with cancer" |
| 9 | Dean(2019)  (ID: 31000351) | -EXCLUDE on "did not focus on self-advocacy experience" |
| 10 | Denyse(2023)  (ID: 37318273) | -EXCLUDE on "did not focus on self-advocacy experience" |
| 11 | Denyse(2022)  (ID: /) | -EXCLUDE on "did not focus on self-advocacy experience"  The literature is from embase and is not included in PubMed, so there is no PMID |
| 12 | Denyse(2021)  (ID: /) | -EXCLUDE on "Conference abstracts, PhD theses, books, commentaries, dissertations or other types of grey literature"  Conference abstract has no PMID |
| 13 | Doherty(2023)  (ID: 37614869) | -EXCLUDE on "did not focus on self-advocacy experience" |
| 14 | Dunn(2018)  (ID: 28145020) | -EXCLUDE on "Conference abstracts, PhD theses, books, commentaries, dissertations or other types of grey literature" |
| 15 | Dvaladze(2020)  (ID: /) | -EXCLUDE on "Conference abstracts, PhD theses, books, commentaries, dissertations or other types of grey literature"  Conference abstract has no PMID |
| 16 | Dvaladze(2020)  (ID: 32348570) | -EXCLUDE on "did not focus on self-advocacy experience" |
| 17 | Ehrlich-Jones(2021)  (ID: 34235506) | -EXCLUDE on "did not focus on self-advocacy experience" |
| 18 | Errico(2006)  (ID: 16430394) | -EXCLUDE on "did not focus on self-advocacy experience" |
| 19 | Garpenhag(2023)  (ID: 36380504) | -EXCLUDE on "did not focus on self-advocacy experience" |
| 20 | Gilkey(2014)  (ID: 24214497) | -EXCLUDE on "Conference abstracts, PhD theses, books, commentaries, dissertations or other types of grey literature" |
| 21 | Gray(1997)  (ID: 9105153) | -EXCLUDE on "did not focus on self-advocacy experience" |
| 22 | Hagan(2017)  (ID: 28060470) | -EXCLUDE on "Conference abstracts, PhD theses, books, commentaries, dissertations or other types of grey literature" |
| 23 | Ho, M. Y(2012)  (ID:/ ) | -EXCLUDE on "did not focus on self-advocacy experience"  The literature is from embase and is not included in PubMed, so there is no PMID |
| 24 | Ho, M. Y(2016)  (ID: 26387559) | -EXCLUDE on "did not focus on self-advocacy experience" |
| 25 | Holtz(2021)  (ID: /) | -EXCLUDE on "did not focus on self-advocacy experience"  The literature is from embase and is not included in PubMed, so there is no PMID |
| 26 | James(2011)  (ID: 21649897) | -EXCLUDE on "did not include population with cancer" |
| 27 | Kahana(2009)  (ID: 20122027) | -EXCLUDE on "Conference abstracts, PhD theses, books, commentaries, dissertations or other types of grey literature" |
| 28 | Katz(2011)  (ID: 21649897) | -EXCLUDE on "did not include population with cancer" |
| 29 | Kissil(2014)  (ID: 24027088) | -EXCLUDE on "did not focus on self-advocacy experience" |
| 30 | Kizub(2019)  (ID: /) | -EXCLUDE on "Conference abstracts, PhD theses, books, commentaries, dissertations or other types of grey literature"  *Conference abstract has no PMID* |
| 31 | Kizub(2020)  (ID:32031438) | -EXCLUDE on "Conference abstracts, PhD theses, books, commentaries, dissertations or other types of grey literature" |
| 32 | Kouspou(2022)  (ID: 32699318) | -EXCLUDE on "Conference abstracts, PhD theses, books, commentaries, dissertations or other types of grey literature" |
| 33 | Kovtun(2008)  (ID: 19058069) | -EXCLUDE on "Conference abstracts, PhD theses, books, commentaries, dissertations or other types of grey literature" |
| 34 | Lamprell(2023)  (ID: 36948549) | -EXCLUDE on "did not focus on self-advocacy experience" |
| 35 | Lythcott(2003)  (ID: 12491496) | -EXCLUDE on "did not include population with cancer" |
| 36 | Mirrielees(2017)  (ID: 26477478) | -EXCLUDE on "did not include population with cancer" |
| 37 | Molina(2016)  (ID: 26891843) | -EXCLUDE on "Conference abstracts, PhD theses, books, commentaries, dissertations or other types of grey literature" |
| 38 | Needles(2018)  (ID: 29610393) | -EXCLUDE on "did not include population with cancer" |
| 39 | P, A. S.(2021)  (ID: 34213358) | -EXCLUDE on "did not focus on self-advocacy experience" |
| 40 | Pedersen(2014)  (ID: 24368241) | -EXCLUDE on "did not focus on self-advocacy experience" |
| 41 | Peitzmeier(2020)  (ID: 31661659) | -EXCLUDE on "Conference abstracts, PhD theses, books, commentaries, dissertations or other types of grey literature" |
| 42 | Pemberton(2020)  (ID: /) | -EXCLUDE on "did not focus on self-advocacy experience"  The literature is from embase and is not included in PubMed, so there is no PMID |
| 43 | Perea(2023)  (ID: 37852716) | -EXCLUDE on "did not include population with cancer" |
| 44 | Ports(2015)  (ID: 25116413) | -EXCLUDE on "did not focus on self-advocacy experience" |
| 45 | Purtzer(2013)  (ID: 22964865) | -EXCLUDE on "did not focus on self-advocacy experience" |
| 46 | Pyke-Grimm(2022)  (ID: 35538622) | -EXCLUDE on "did not include population with cancer" |
| 47 | Rechis(2013)  (ID: 23229180) | -EXCLUDE on "Conference abstracts, PhD theses, books, commentaries, dissertations or other types of grey literature" |
| 48 | Ridner(2016)  (ID: 26810422) | -EXCLUDE on "did not focus on self-advocacy experience" |
| 49 | Roche(2016)  (ID: 27601509) | -EXCLUDE on "did not focus on self-advocacy experience" |
| 50 | Sawa(2013)  (ID: /) | -EXCLUDE on "did not focus on self-advocacy experience"  The literature is from embase and is not included in PubMed, so there is no PMID |
| 51 | Sneha(2022)  (ID:35667920 ) | -EXCLUDE on "did not include population with cancer" |
| 52 | Tergas(2016)  (ID: /) | -EXCLUDE on "did not focus on self-advocacy experience"  The literature is from embase and is not included in PubMed, so there is no PMID |
| 53 | Thomas(2023)  (ID: 35794411) | -EXCLUDE on "did not include population with cancer" |
| 54 | Tideman(2015)  (ID:25819844 ) | -EXCLUDE on "did not include population with cancer" |
| 55 | Trump(2011)  (ID: 20978956) | -EXCLUDE on "did not include population with cancer" |
| 56 | Walling(2022)  (ID: /) | -EXCLUDE on "did not include population with cancer"  The literature is from embase and is not included in PubMed, so there is no PMID |
| 57 | Welten(2022)  (ID: /) | -EXCLUDE on "did not focus on self-advocacy experience"  The literature is from embase and is not included in PubMed, so there is no PMID |
| 58 | Zebrack(2006)  (ID: 16482447) | -EXCLUDE on "did not include population with cancer" |
| Second search results | | |
| 1 | McCully(2024)  (ID: 39021547) | -EXCLUDE on "did not focus on self-advocacy" |
| 2 | Iannarino (2024) (ID:39663816) | -EXCLUDE on "did not focus on self-advocacy" |
| 3 | Thomas (2023) (ID:37243943 ) | -EXCLUDE on "Conference abstracts, PhD theses, books, commentaries, dissertations or other types of grey literature" |
| 4 | Dave (2024) (ID:39329173) | -EXCLUDE on "did not focus on self-advocacy experience" |
| 5 | Krishnamurthy (2024) (ID:38957483) | -EXCLUDE on "did not focus on self-advocacy experience" |
| 6 | Rogers (2023) (ID:37619592 ) | -EXCLUDE on "did not focus on self-advocacy" |
| 7 | Torres (2024) (ID:38673406 ) | -EXCLUDE on "did not focus on self-advocacy experience" |
| 8 | Denyse (2023) (ID:37318273) | -EXCLUDE on "did not focus on self-advocacy experience" |
| 9 | Noronha (2024) (ID:39157506) | -EXCLUDE on "did not focus on self-advocacy experience" |
| 10 | Otty (2023) (ID:36710377) | -EXCLUDE on "did not focus on self-advocacy" |
| 11 | Garpenhag (2023) (ID:36380504) | -EXCLUDE on "did not focus on self-advocacy" |
| 12 | Thomas (2024) (ID:39411999) | -EXCLUDE on "did not include population with cancer" |
| 13 | Thomas (2023)  (ID: 35794411) | -EXCLUDE on "did not focus on self-advocacy experience" |
| 14 | Felder (2024)  (ID:/) | -EXCLUDE on "Conference abstracts, PhD theses, books, commentaries, dissertations or other types of grey literature" *Conference abstract has no PMID* |
| 15 | Wilkenfeld (2023) (ID:36377241) | -EXCLUDE on "did not focus on self-advocacy" |
| 16 | Xue gong (2024) (ID:36377241) | -EXCLUDE on "did not focus on self-advocacy" |
| 17 | Mengya Guo (2024) (ID:36377241) | -EXCLUDE on "did not include population with cancer" |
