# Supplementary material for: The experience of self-advocacy among cancer patients: A qualitative meta-synthesis
Source: PLoS One. 2025 Apr 16;20(4):e0321719. doi: 10.1371/journal.pone.0321719 (PMC12002448; doi:10.1371/journal.pone.0321719)
Supplement: S6 Appendix — (DOCX) [file pone.0321719.s006.docx]

**S5 Appendix：Table 1 Characteristics of included studies**

| Study | Aim | Demographic | | Methods | |  | Results |
| --- | --- | --- | --- | --- | --- | --- | --- |
|  |  | N | Sample | Methodology | Data collection | Data analysis |  |
| Sarah Bell et al(2023) | Describe the self-advocacy experiences | 10 | advanced breast or gynecologic cancer | descriptive  qualitative study | semi-structured  interview | Content analysis | 1.speaking up and speaking out；  2.interacting with the healthcare team；  3.relying on support from others |
| Hagan et al(2013) | Explore ovarian cancer survivors’ experiences of self-advocacy | 13 | ovarian cancer survivors | phenomenon study | in-depth  interviews | Content analysis | 1.knowing who I am and keeping my psyche intact;  2.knowing what I need and fighting for it. |
| Thomas et al(2022) | To describe the key components of self-advocacy among men with cancer. | 28 | adult men with a history of invasive cancer | descriptive  qualitative study | semi-structured interview | Content analysis | 1.managing through information and planning;  2.finding the best team and falling in line;  3.strategic social connections |
| Thomas et al(2023) | The participant perspectives of a novel, self-advocacy serious game intervention | 40 | women with  advanced cancer | qualitative  study | one-on-one  interviews | Content analysis | 1.overall acceptability；2.seeing myself in most scenarios and wanting more content；3.giving me the go ahead to expect more；4.offering ideas for how to stand up for myself；5.reinforcing what I am already doing；6. reminding me of what I have. |
| Sydney et al(2017) | To engage survivor–advocates by describing their experiences living with lung cancer | 19 | patients with lung cancer | qualitative  study | one-on-one  interviews | Narrative analysis | Stage I. Live: The stage “Live” refers to survivors’ personal lived experience with lung cancer themselves；  Stage II.learn:Knowledge gives me a future addresses the importance of accessing knowledge and ways to approach this；Learning to be empowered in my care；Finding my way through the system；  Stage III.pass it on：Pulling lung cancer out of the shadows；The urgency of sharing stories；Showing the way involved becoming a guide to other patients；The secret handshake of community was also vital |
| Hagan et al(2016) | To explore the language of self-advocacy | 13 | woman  cancer Survivors | qualitative  study | focus group | Discourse analysis | 1.maintaining a positive attitude  2.needing and being scared of information  3.connection with health care team |
| Zhirong Jiang et al(2023) | To gain an in-depth understanding of the self-advocacy experience of breast cancer patients | 20 | woman with breast cancer | phenomenon  study | semi-structured interview | Colaizzi’s Method | 1.weak awareness of self-advocacy  2.multiple factors on self-advocacy motivation  3.challenges in the process of self-advocacy  4.positive experience of self-advocacy |
