# Supplementary material for: The experience of self-advocacy among cancer patients: A qualitative meta-synthesis
Source: PLoS One. 2025 Apr 16;20(4):e0321719. doi: 10.1371/journal.pone.0321719 (PMC12002448; doi:10.1371/journal.pone.0321719)
Supplement: S7 Appendix — (DOCX) [file pone.0321719.s007.docx]

**S7 Appendix: Results of CASP quality appraisal**

|  | 1 | 2 | 3 | 4 | 5 | 6 | 7 | 8 | 9 | 10 | **Total Score** |
| --- | --- | --- | --- | --- | --- | --- | --- | --- | --- | --- | --- |
| Sarah Bell et al 2023 | Y | Y | Y | Y | Y | Y | Y | Y | Y | Y | **10** |
| Hagan et al 2013 | Y | Y | Y | Y | Y | C | N | Y | Y | Y | **8** |
| Thomas et al 2022 | Y | Y | Y | Y | Y | C | Y | Y | Y | Y | **9** |
| Thomas et al 2023 | Y | Y | Y | Y | Y | C | N | Y | Y | Y | **8** |
| Sydney et al 2017 | Y | Y | Y | C | Y | C | N | Y | Y | Y | **7** |
| Hagan et al 2016 | Y | Y | Y | Y | Y | N | N | Y | Y | Y | **8** |
| Zhirong Jiang et al 2023 | Y | Y | Y | Y | Y | C | Y | Y | Y | Y | **9** |

Y，yes；N，no；C，can’t tell.

1.Is there a clear statement of the aim of the research?

2.Is a qualitative methodology appropriate?

3. Is the research design appropriate to address the aims of the research?

4Is the recruitment strategy appropriate to the aims of the research?

5.Is the data collected in a way that addressed the research issue?

6.Has the relationship between researcher and participants been adequately considered?

7.Have ethical issues been taken into consideration?

8.Is the data analysis sufficiently rigorous?

9. Is there a clear statement of findings?

10.How valuable is the research?
