# Supplementary material for: The experience of self-advocacy among cancer patients: A qualitative meta-synthesis
Source: PLoS One. 2025 Apr 16;20(4):e0321719. doi: 10.1371/journal.pone.0321719 (PMC12002448; doi:10.1371/journal.pone.0321719)
Supplement: S8 Appendix — (DOCX) [file pone.0321719.s008.docx]

**S8 Appendix: Themes, sub-themes, original descriptive theme, and illustrative quotations**

| Theme | Sub-theme | Original descriptive theme | Illustrative quotations from participants |
| --- | --- | --- | --- |
| Benefits | Gain confidence | Face the disease with a positive attitude | *“I can’t sit here and wallow in self-pity because then I start getting depressed and, if I get depressed, I don’t do things, I can’t help my kids. I mean, I just can’t do anything”*Hagan, T. L., & Donovan, H. S. (2013)  *“As soon as you go and put yourself in that negative mode, the cancer’s going to take over. So that’s how I get myself back out of it”*Hagan, T. L., & Donovan, H. S. (2013)  *“ it allows her to fight, keep a sense of selfworth, and believe that she can overcome the cancer and “never go down.”*Hagan, T. L., & Medberry, E. (2016)  *“you know you fight for all you’re worth.you never go down.you know what I mean?”*Hagan, T. L., & Medberry, E. (2016)  *“I have people in my family that are very educated and in the medical field, nurses. And we’ve been through cancer with my mom and my dad and my sister. And I have a huge family, and so I think someone that is not as lucky as I am to have that big of a family and that educated of a family, I think they will benefit from it.”*Thomas, T. H. (2023)  *“I never knew that I could interrupt a doctor to ask a question, to add a new direction to my care.”*Thomas, T. H. (2023)  *“I wish there would have been more of the scenarios because I got done with them relatively quickly, went back, redid them over again and redid them a couple of times.”*Thomas, T. H. (2023)  *“Just check out when really a little bit hard ah, after meeting these patients, we communicate with each other, they advised me, called me not to be afraid, this is not what, my mood slowly better.”*Zhirong Jiang(2023)  *“I also like to say to patients, like the ones we played well in it, I did a lot of ideological work with them. All of us added Wechat, so we said that we had this disease and faced it bravely together”*Zhirong Jiang(2023)  *“I had been hearing online from people throughout the year about a new mutation called ROS1 ... I gave them permission to test ...I had a very, very strong indication of a ROS1 mutation.Three days later I’ve flown out [of state]. I took my first pill ... And my very first scan there was no evidence of disease and have been for 28 months.”*Dy, S. M.(2017)  *“They had issues that I am not experiencing. But at the same time, I may down the road. So, it was good to know what might be coming as far as other people’s experiences”*Thomas, T. H. (2023) |
|  | Improve self-management ability | Acquire sufficient knowledge about disease | *“I’m reading actual medical newsletters, from reputable places”*Bell, S.(2023)  *““And I’ve gotten a little more proactive at the doctor, just asking more and more questions”*Bell, S.(2023)  *“Feeling confident that I’m a patient with certain needs, and I’m not going to be overly forceful of that, and I’m not going to bump somebody else out of line to get what I want, but I am going to make it clear that this is something that I want to get, so that’s what I did”*Bell, S.(2023)  *“They're going to put me on this new inhibitor program and I'm reading more about it; I got all the details on it.”*Hagan, T. L., & Donovan, H. S. (2013)  *“Alright so at that point so they-they want me to get the pelvic and the abdomen.I says “but for this plan, it says you should also get the chest one.so why don’t I get all three of them done now instead of like waiting”Hagan, T. L., & Medberry, E. (2016)*  *“But in that course of time, I actually read three or four books on prostate cancer…. [My care] was pretty much standard, what the book says and what my recovery was pretty much the same”*Thomas, T. H. (2022)  *“They were very responsive. We actually worked out a plan…. And this was all done back and forth beginning that week by phone, so it was very, very good”*Thomas, T. H. (2022)  *“Yeah, I have a lot of activities I used to do I cut back on. Yeah, it’s a different way of life…. And one day, one thing will be hurting. The next day, something else will hurting. It’s always something different each day… So you figure out a plan. Okay. Now, I’ll get up and walk around see if I can do that first. And then you just move on from there.”*Thomas, T. H. (2022)  *“It made me think about privilege a lot and who is able to afford to not work and still pay rent and do all these things. And so it made me really think about other people’s experience a lot more than my own because I’m pretty lucky and I have a really strong support system. And so it just*  *kind of made me mad that a lot of people don’t have that. And that even though it was hard for me, it’s astronomically harder for other people to make those choices”*Thomas, T. H.(2023)  *“It’s comforting. It helps you decide things about whether to go out, whether to run, whether to go shopping, call your doctor, all of the above. So, for me, it was like an outline of”*Thomas, T. H.(2023)  *“It kind of reinforced things, what you should or should not do. And really nice to help others if they want it.”*Thomas, T. H.(2023)  *“I know now more than ever that I really am the person who cares the most about my future, even with all these great people who are concerned about me.”*Dy, S. M.(2017)  *“I’m very lucky to be cancer-free. So I do not need treatments right now, but I am always interested in seeing, what are those drugs that may be available to me in the future, in case ...I mean I know the reality that lung cancer, you know, can come back.”*Dy, S. M.(2017)  *“I approach my doctor and tell him what I’m thinking and get his feedback, if he disagrees, and so I advocate for what I feel is the best treatment available.”*Dy, S. M.(2017) |
|  | Interaction and share | Share self-advocacy experiences with others | *“I did a lot of ideological work for several patients who were playing well”*Zhirong Jiang(2023)  *“Wherever I can—I feel somebody that has survived—somebody that has lived longer than the average, I feel obligated in my role to stand up and speak from as high a perch as I can possibly speak”*Dy, S. M.(2017)  *“It’s really about trying to get those people out of the dark or out of the loneliness of the disease so that they can be advocates.”*Dy, S. M.(2017)  *“I’m hopeful that there will be a true outpouring, nationwide,of compassion for people with lung cancer, like an awakening so to speak, enlightenment, of, “Hey, these people do matter”.”*Dy, S. M.(2017)  *“Like they didn’t know where to begin, it’s like you’re on an airplane, you’re dropped in a foreign country, you don’t speak the language. So what I did, “Okay, here’s what you start with.”Because patients, and their families especially, need some kind of control. ”*Dy, S. M.(2017) |
| Challenges | Lack of awareness | weak awareness of self-advocacy | *“Usually the body has any discomfort, the doctor asked me I will speak, don't ask me I will not speak." I don't think this discomfort needs to be resolved, it is a normal reaction to the disease”*Zhirong Jiang(2023)  *“I have no strength in chemotherapy, very uncomfortable, they (patients) that is to say the needle back to have a response, I did not tell the doctor, only their own slowly overcome ah, this is the reaction of chemotherapy, what method to solve it (sigh).”*Zhirong Jiang(2023)  *“Now that I have a disease, the doctor says it is better to treat it. I have never had this disease, and I do not know whether the treatment plan is good or not. He said that this plan is good, so let's follow it.”*Zhirong Jiang(2023) |
|  | Obstacles | Barriers to self-advocacy | *“I think releasing that responsibility and putting it on a physician, somebody that’s knowledgeable, is a relief.”*Bell, S.(2023)  *“Now that I have had chemotherapy, all my hair has fallen out, and a breast has been removed. I feel very inferior and am unwilling to deal with others, and I am unwilling to tell anyone about my situation.”*Zhirong Jiang(2023)  *“I don't know how to treat it later. It was all my children who went to communicate with the doctor, but they didn't tell me. My two children said you didn't need to listen to this and told me not to inquire about it”*Zhirong Jiang(2023)  *“During the ward rounds yesterday morning, I wanted to ask a few questions, but the doctor said directly: 'Don't talk about anything else. You just need to answer my questions. You can only answer yes or no.' I am speechless.”*Zhirong Jiang(2023)  *“To be honest, sometimes we're afraid to say what's on our minds... Afraid the doctor has a problem with us, you don't follow the doctor's order is not cooperating with the doctor”*Zhirong Jiang(2023)  *“What's the problem? You ask twice, the doctor gets impatient”*Zhirong Jiang(2023) |
| External environmental support | health system support | Connection with health care team | *“I need her guidance, but I just also need her to be completely honest with me about what’s happening, and she’s doing that”*Bell, S.(2023)  *“Three participants cited honesty from their care team as being a key component to good care.”*Bell, S.(2023)  *“Dr. X . . . could have been more open. . . . But first, in the beginning, they could have, I don’t know, been a little more open about stuff and tell me more”*Bell, S.(2023)  *“Let somebody else that knows what the heck they’re talking about make that decision. Why am I making that decision when I don’t know what the heck I’m talking about? . . . I think releasing that responsibility and putting it on a physician, somebody that’s knowledgeable, is a relief. And I don’t have to carry that burden of should I—let them [inaudible]. And I think, really, that’s how it should be”*Bell, S.(2023)  *“if I really need to know something the man’s gonna to tell me.So I really suck at that.”*Hagan, T. L., & Medberry, E. (2016)  *“I have a friend who is actually an oncologist. And so I was consulting her. And so I do not know what I would have done without her, to tell you the truth. Because when you get all of these conflicting opinions – like I said, I’ve been through, basically, five, maybe six opinions”*Thomas, T. H.(2022)  *“If I had two people to choose from, and one was better at the bedside manner and the other one was better at the medical side, I would take the medical side.”*Thomas, T. H.(2022)  *“Finding a doctor I could talk to, and who would give me answers, was my purpose in life after 2 or 3 visits to the first clinic I visited”*Thomas, T. H.(2022)  *“He said he had a lot of tricks. He said, ‘If one thing does not work, we’ll try something else.’ He said, ‘What was true 5 years ago in this field is not true now.’ And he said, ‘We’re learning new things every day.’ And he said, ‘We’re going to get started.’ Then I said, ‘When?’ I figured maybe a month. He said, ‘Tomorrow.’ So I went right in and we started up”*Thomas, T. H.(2022) |
|  | social support | Social connection | *“I did need her voice and her ears and her eyes and her strength. Because we have no other family in the area, so it’s just my husband and I.”*Bell, S.(2023)  *“I have an incredible support system. My wife and I have 2 kids, and I have a very not huge but deep circle of friends and colleagues. So work has been wonderful, friends have been wonderful, family has been wonderful, and I really don’t have any physical limitations, at this point, that keep me from doing things”*Bell, S.(2023)  *“But people came surprisingly up to me when I was first diagnosed to let me know, ‘We’re here, and we can go with you to get a wig. Don’t worry. We’ve all been through this.’ People that I never even knew had breast cancer that told me other people that I never knew either that had breast cancer. So I’m not lacking in a social network”*Bell, S.(2023)  *“Believe me, my employer has been more than generous and willing to assist me, particularly with doctors’ appointments that have come up unexpected”*Bell, S.(2023)  *“One of the issues is always how much to tell and who to tell… I did not tell my wife anything until that time came. And I think that was probably the best decision…. It’s your information to share or not share because then sometimes, you feel like you have to manage how people are responding, which for managing work situations is different from managing the emotional side even your family members or close friends are going to have.”Thomas, T. H.(2022)* |
