# Supplementary figures and images for: The experience of self-advocacy among cancer patients: A qualitative meta-synthesis
Source: PLoS One. 2025 Apr 16;20(4):e0321719. doi: 10.1371/journal.pone.0321719 (PMC12002448; doi:10.1371/journal.pone.0321719)

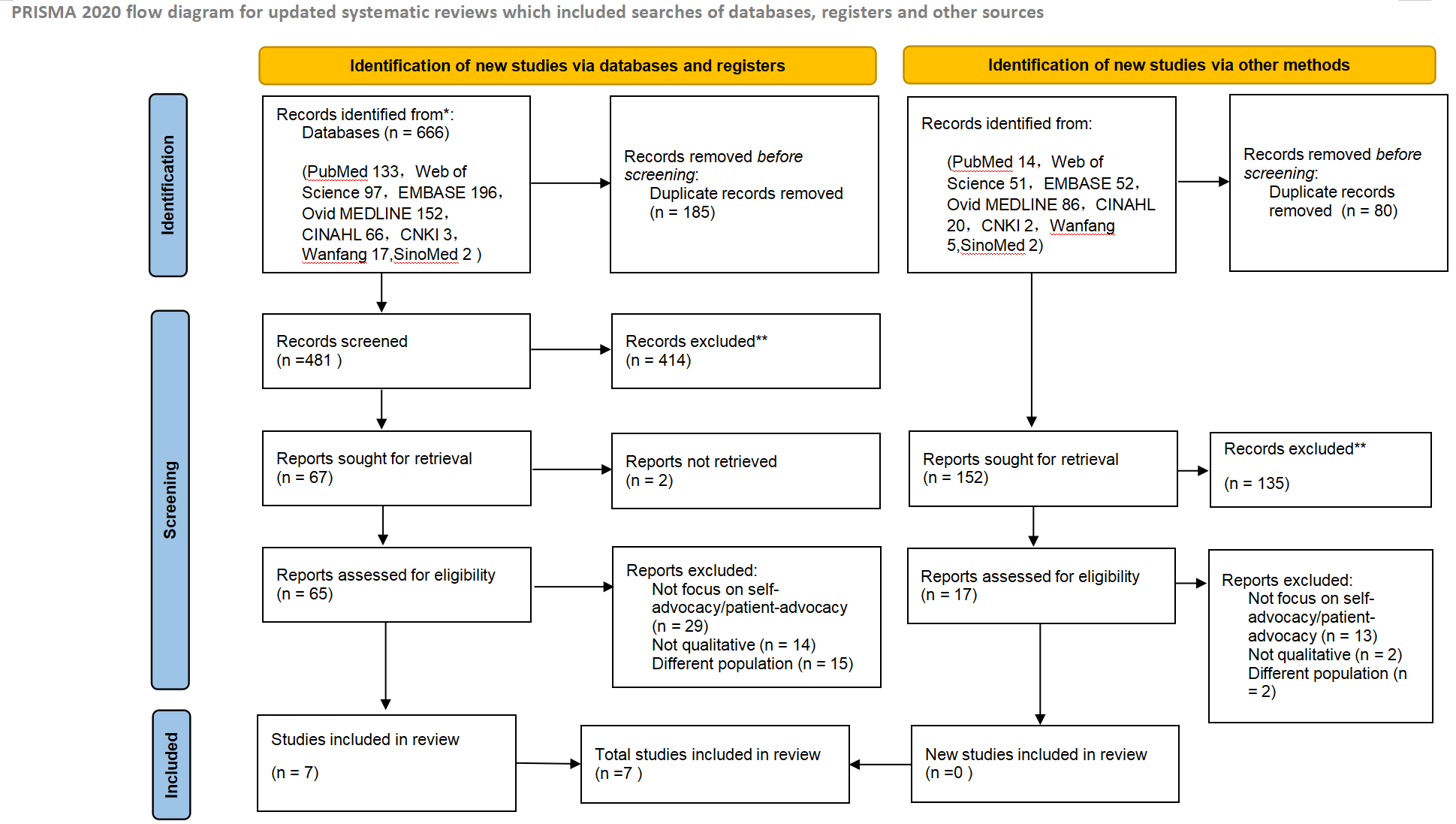

Supplement: S1 Fig — (TIF) [file pone.0321719.s009.tif]

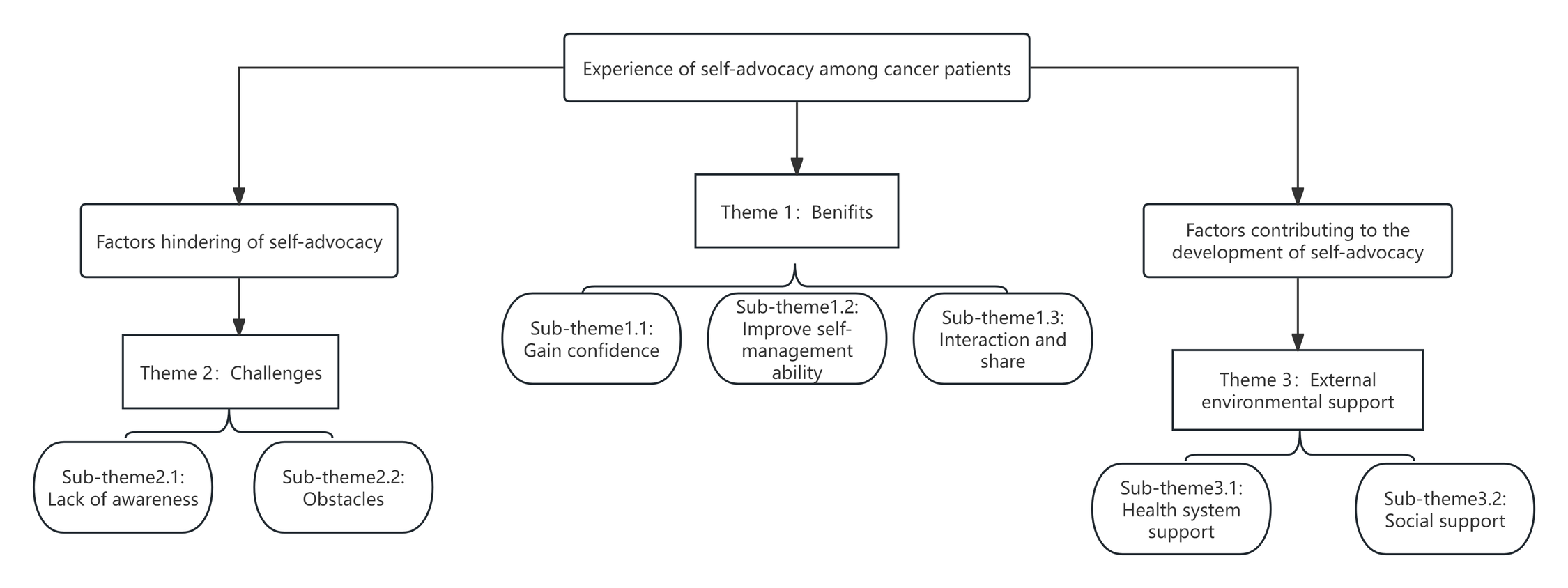

Supplement: S2 Fig — (TIF) [file pone.0321719.s010.tif]
